# Mysterious Keratitis Responding Favorably to Antiviral Therapy

**DOI:** 10.7759/cureus.66384

**Published:** 2024-08-07

**Authors:** Surbhi A Chodvadiya, Megha R Kotecha, Varsha Manade, Gufran A Kamdar, Jessica Sangwan

**Affiliations:** 1 Department of Ophthalmology, Dr. D. Y. Patil Medical College, Hospital & Research Centre, Pune, IND

**Keywords:** vzv, hsv, corneal ulcer, antiviral therapy, keratitis

## Abstract

Keratitis, characterized by inflammation of the cornea, presents a diagnostic challenge, particularly when the etiology remains elusive. Here, we report a perplexing case of keratitis in a 35-year-old patient with no identifiable risk factors or predisposing conditions. Despite the initial uncertainty, empirical treatment with antiviral medications led to a rapid resolution of symptoms and improvement in corneal health. This case underscores the importance of considering viral etiologies even in cases with atypical presentations and highlights the potential efficacy of antiviral therapy in such scenarios. Further investigation is needed to understand the underlying causes and improve treatment approaches for similar cases of unexplained keratitis.

## Introduction

Keratitis, a common eye condition, often leads to partial or complete vision loss [1]. It is influenced by both infectious agents like bacteria, fungi, Acanthamoeba, and viruses, and non-infectious factors such as eye trauma, chemical and UV exposure, and contact lens use. Viral infections, in particular, are a major cause of corneal clouding [2]. Necrotising stromal viral keratitis is a rare but serious condition characterized by inflammation and necrosis of the corneal stroma caused by viral infection, often herpes simplex virus (HSV) or varicella-zoster virus (VZV) [3]. Typical symptoms include severe eye pain, redness, photophobia, and decreased vision. Diagnosis involves a clinical examination, corneal scrapings for viral culture or polymerase chain reaction (PCR), and sometimes confocal microscopy. Treatment usually involves antiviral medications, corticosteroids, and supportive measures like lubricating eye drops [4]. However, prompt diagnosis and management are crucial to prevent vision-threatening complications such as corneal perforation and scarring.

## Case presentation

**Figure 1:**
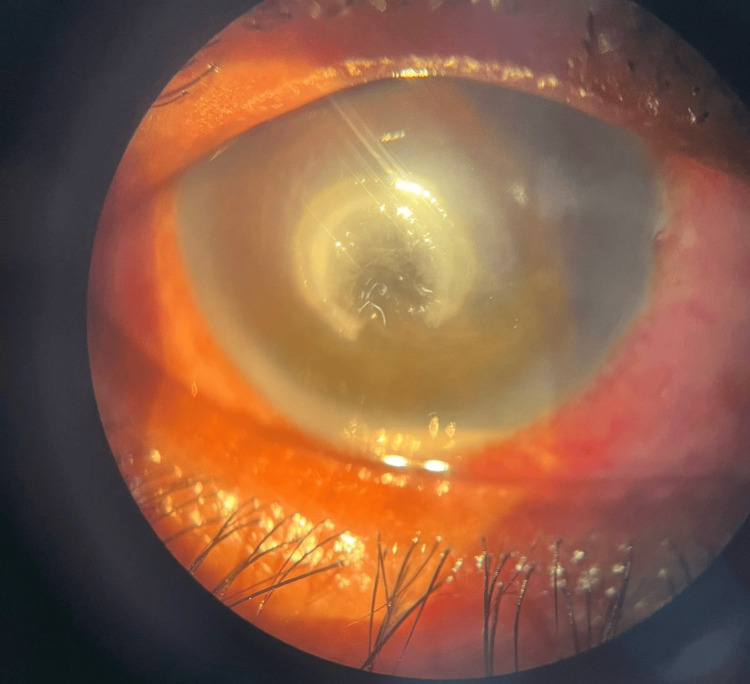
Day 1 slit-lamp picture The patient's right eye showed diffuse circumciliary congestion, a central corneal ulcer in a horseshoe shape, and a Descemet’s membrane fold around the ulcer. Infiltrates were observed in a ring shape surrounding the ulcer, with a hypopyon present in the anterior chamber.

**Figure 2:**
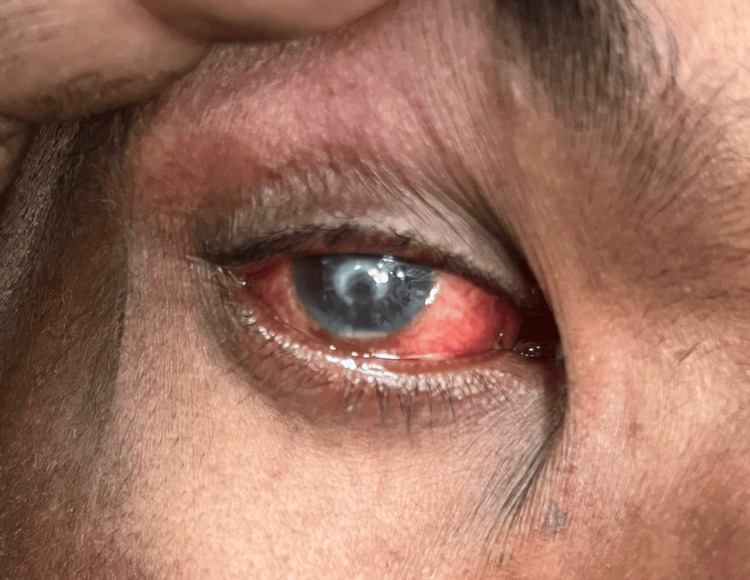
Day 1 naked eye picture Visible redness, circumciliary congestion, and central corneal ulcer were observed in the right eye without the use of a slit lamp.

A 35-year-old man from Pune, who works in the field of construction, reported redness, pain, and sensitivity to light in his right eye. An examination showed widespread circumciliary congestion in the conjunctiva of the anterior segment. A central corneal ulcer, measuring approximately 4×4 mm in a horseshoe shape, was observed, accompanied by a Descemet’s membrane fold around the ulcer. Additionally, infiltrates were noted in a ring shape surrounding the ulcer, with a 1-mm hypopyon present inferiorly in the anterior chamber (Figures 1, 2). There was no history of foreign body entry or exposure to water bodies or contact lens use.

**Figure 3:**
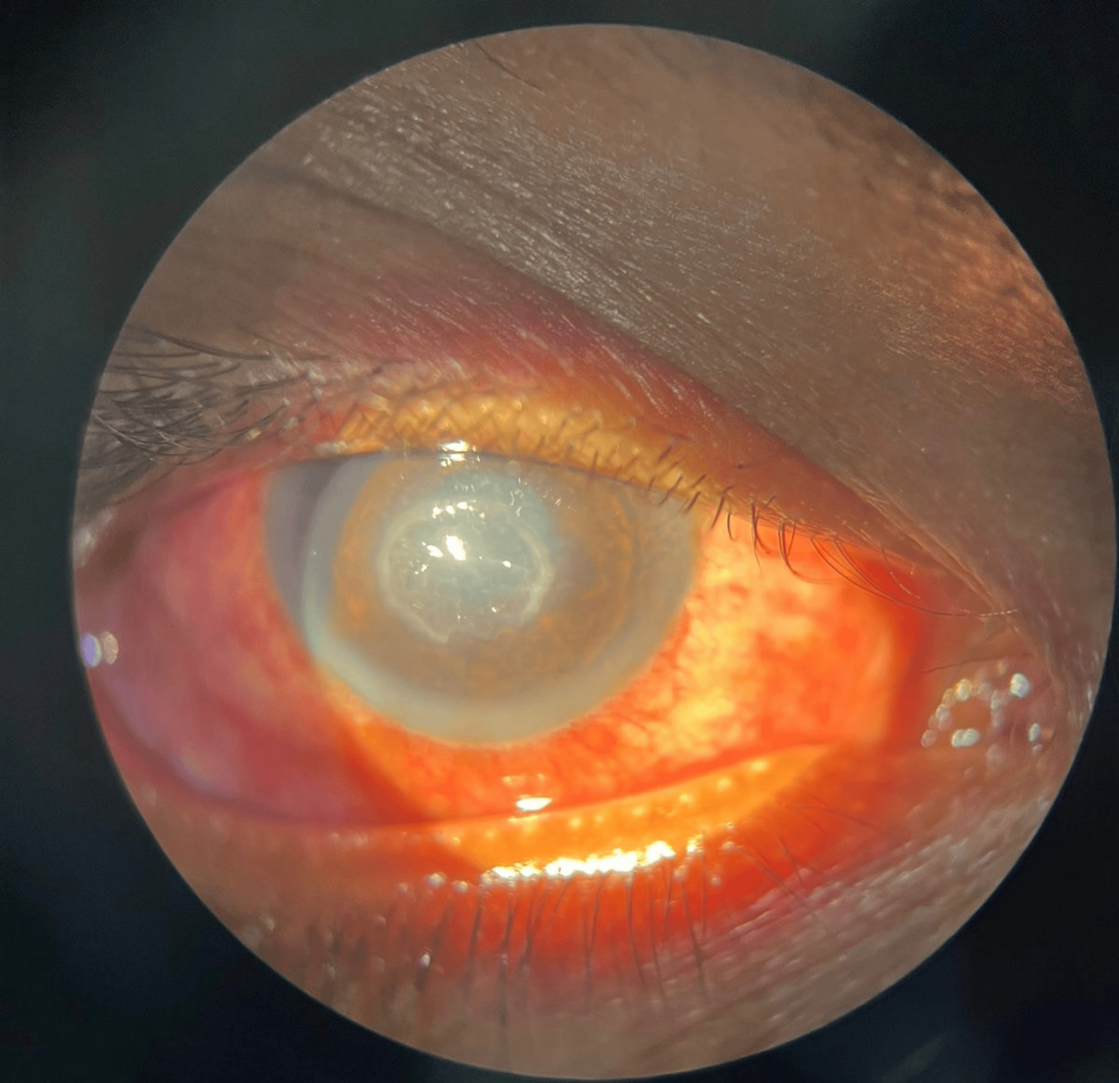
Day 7 slit-lamp picture The patient's right eye showed notable healing progress, with reduced inflammation and improved clarity of the corneal ulcer.

The patient was promptly initiated on empirical treatment, including topical antibiotic eye drops, cycloplegic eye drops, and eye ointment (chloramphenicol) at night [5]. Following this regimen, the patient's vision showed improvement. Despite initial treatment, corneal scraping cultures tested negative for any microorganisms, including specific checks for bacterial and fungal infections using Gram and KOH staining [6]. Subsequently, the patient was prescribed oral acyclovir (400 mg twice a day) for a duration of five days. Remarkably, the patient responded favorably to this therapeutic intervention, experiencing significant alleviation of symptoms (Figure 3). The patient was followed up on a weekly basis.

**Figure 4:**
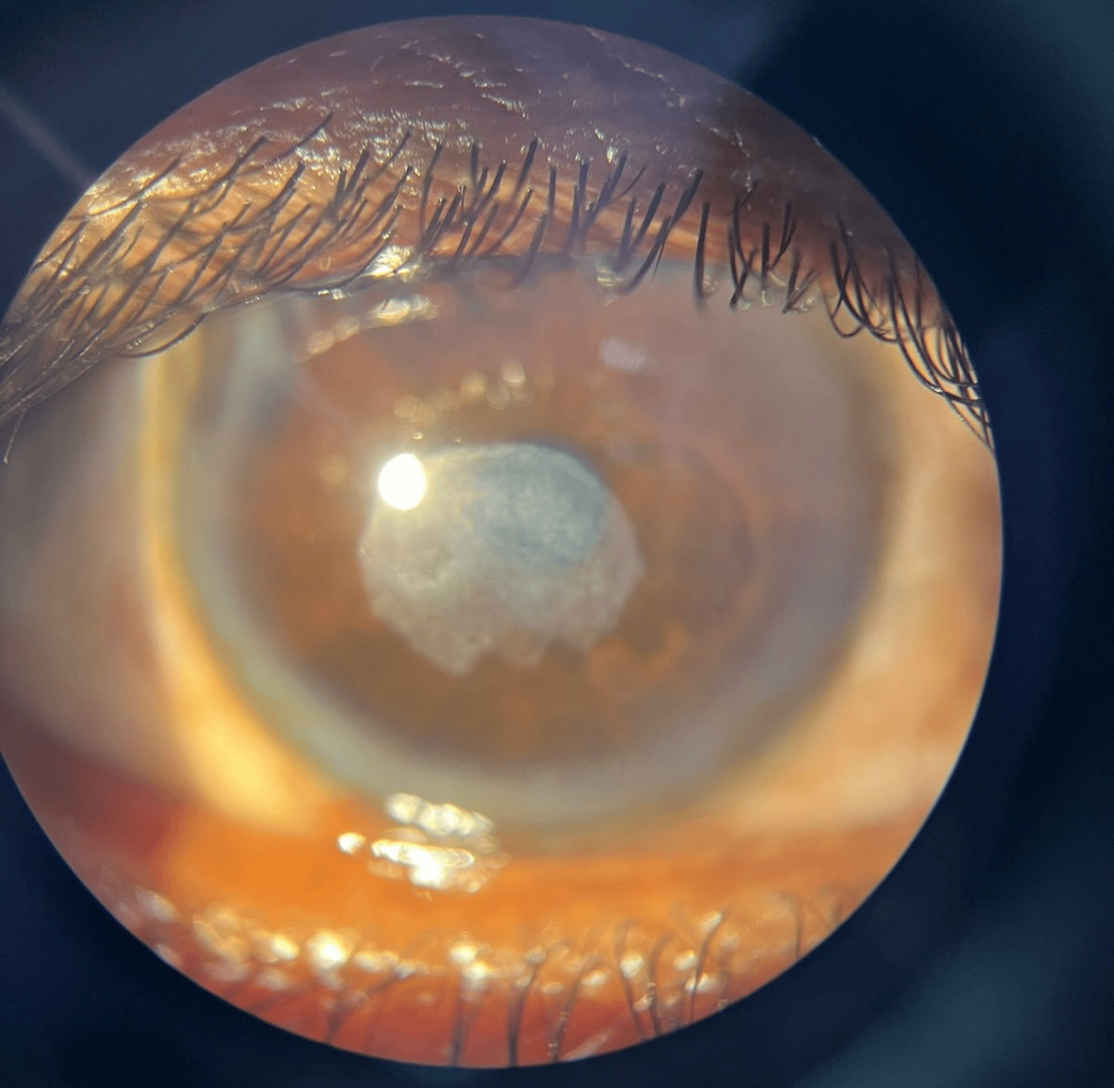
Day 14 slit-lamp picture post-antiviral therapy A significant healing of the central corneal ulcer was noted with restored corneal clarity and resolved infiltrates.

To further enhance ocular comfort and promote healing, lubricating eye drops were incorporated into the treatment regimen [7]. The patient reported increased comfort and satisfaction with the modified treatment approach. Overall, the patient demonstrated remarkable improvement and resolution of symptoms following the comprehensive management strategy, highlighting the efficacy of antiviral therapy in the absence of an identifiable microbial etiology (Figure 4).

## Discussion

The case presented here underscores the diagnostic and therapeutic challenges encountered in managing ocular pathologies, particularly in cases of keratitis with atypical presentations. The patient's line of occupation, i.e., construction industry, potentially exposed him to various environmental factors, predisposing him to corneal injury and subsequent infection. However, the absence of identifiable risk factors or predisposing conditions initially complicated the diagnostic process [8].

The clinical presentation, characterized by diffuse circumciliary congestion, central corneal ulcer, Descemet's membrane fold, and infiltrates with hypopyon, raised suspicion for infectious keratitis. The prompt initiation of empirical treatment with topical antibiotics and cycloplegics aimed to mitigate the risk of microbial proliferation and alleviate symptoms. Despite empirical treatment, corneal scraping cultures returned negative, indicating a challenging diagnostic dilemma [9].

In the absence of microbiological confirmation, the decision to initiate oral acyclovir therapy was based on the clinical suspicion for viral etiology. The patient's remarkable response to antiviral therapy suggests a potential viral component contributing to the keratitis, despite negative culture results. This observation highlights the importance of clinical judgment and therapeutic trial in managing elusive cases of keratitis [10,11].

## Conclusions

The effective management of this case highlights the significance of a multidisciplinary approach that includes clinical expertise, diagnostic methods, and tailored therapeutic interventions. Although the initial cause of the keratitis was unclear, the combination of empirical treatment with topical antibiotics and subsequent oral antiviral therapy led to a marked improvement in symptoms and corneal health. This case illustrates the potential benefits of antiviral therapy for keratitis with a suspected viral origin, even without microbiological confirmation. Further research is necessary to better understand the role of antiviral treatments in managing viral keratitis and to refine treatment strategies for similarly challenging cases. Continued vigilance and careful monitoring are crucial to prevent complications and ensure positive outcomes in patients with complex eye conditions.
